# Stakeholders’ perspectives on barriers and facilitators to implementing extra physical activity in secondary schools to improve adolescents' health and academic performance

**DOI:** 10.3389/fspor.2025.1524414

**Published:** 2025-02-20

**Authors:** Susanne Andermo, Lisette Farias, Björg Helgadóttir, Örjan Ekblom, Gisela Nyberg

**Affiliations:** ^1^ Department of Neurobiology, Care Sciences and Society, Division of Nursing, Karolinska Institutet, Huddinge, Sweden; ^2^ Department of Physical Activity and Health, The Swedish School of Sport and Health Sciences (GIH), Stockholm, Sweden; ^3^ Department of Neurobiology, Care Sciences and Society, Division of Occupational Therapy, Karolinska Institutet, Huddinge, Sweden; ^4^ Department of Clinical Neuroscience, Karolinska Institutet, Stockholm, Sweden; ^5^ Department of Global Public Health, Karolinska Institutet, Stockholm, Sweden

**Keywords:** physical activity, schools, adolescents, health promotion, qualitative research

## Abstract

**Introduction:**

There is an association between physical activity and both health and academic performance. However, there is still a lack of consensus on how to engage adolescents in physical activity interventions in secondary schools. One approach to better understand the activities and strategies supporting effective implementation is to involve school staff and adolescents in the early stages of planning and preparing for interventions. Therefore, the aim of this study is to explore how multiple stakeholders, including school staff, students, and experts, perceive the barriers and facilitators for implementing a school-based intervention that extends the school day with additional physical activity in Sweden.

**Material and methods:**

This inductive explorative qualitative study involved 16 participants. Three focus groups with school staff, including principals (*n* = 3), teachers (*n* = 6), and students (*n* = 4), and three interviews with experts were conducted. The planned intervention and its components were presented to the participants in the focus group and interviews to discuss them based on their previous experiences and thoughts of implementing physical activities or health promotion programmes in their schools or with adolescents. Data were analysed using qualitative content analysis.

**Results:**

Three categories emerged: (1) “types of activities offered”, highlighting the importance of designing activities that are fun, inclusive and unusual; (2) “integration of the activities into school curriculum” to promote sustainability of the intervention and incorporate physical activity throughout the school day and across school subjects, and (3) “Management support and funding” referring to the funded time and facilities that teachers leading the activities need to facilitate implementation.

**Conclusions:**

Before implementing extra physical activity in a school setting, it is important to understand what activities enhance students' motivation and the type of support teachers need from the school principal and administration. This includes funding for teachers' time, appropriate scheduling of the activities, and access to facilities.

## 1 Introduction

Given that adolescents spend a significant proportion of their time in school, it has been suggested that schools are ideal for implementing physical activity interventions (1, 2). Physical activity has been shown to benefit adolescents' physical and mental health and support their academic performance (3–6). Recent publications from Nordic countries (7–9) also show that additional physical activity contributes to youth's physical health and improves their grades. Despite the known benefits of physical activity, a concerning trend persists where approximately 70%–80% of adolescents aged 11–17 globally and in Sweden do not reach the recommendations for daily physical activity (10, 11). Furthermore, adolescents from low socioeconomic backgrounds are less active and have fewer opportunities for physical activities than their peers from high socioeconomic backgrounds (11). The disparities in physical activity levels highlight the need for targeted interventions within school settings to ensure equitable access to physical activity opportunities for all adolescents, regardless of socioeconomic status (12).

Implementing physical activity interventions in the school context has gained attention as a strategy to improve adolescents' health and academic performance. Despite the widespread implementation of these initiatives, previous research lacks consensus regarding the most effective strategies for successful implementation, particularly concerning types of physical activity and organisational factors (13, 14). Recent systematic reviews have highlighted that many school-based physical activity interventions fail to enhance daily physical activity in this context (14). More research is needed to strengthen the understanding of the factors contributing to effective implementation. One of the primary challenges identified is that many school-based interventions do not adequately consider the needs of the students and the specific contextual conditions in which they are implemented (15). This is particularly concerning given the findings of Jago et al. (16), which emphasise the importance of tailoring interventions to address local contexts and the diverse needs of students to enhance effectiveness and sustainability. This approach encourages a shift from rigid, uniform intervention designs to more flexible strategies, allowing schools to adapt the content of physical activity programmes to their unique circumstances (16). Furthermore, van Sluijs et al. (17) argue that although schools are essential arenas for health promotion, there is a need to consider the schools' policies and social and physical environment. A study by McHale et al. (18) supports this view, highlighting the importance of understanding organisational and interpersonal factors that influence implementation. As Mc Mullen et al. (19) highlight, schools often have other competing priorities and time constraints that hinder effective implementation. Thus, there is a need for more research before implementing school-based physical activity interventions for adolescents to understand better organisational factors and how to best design such interventions, especially in lower secondary schools (20).

Understanding the factors that facilitate successful implementation is crucial but challenging due to the complexity of school settings (21–23). To address this complexity, the co-development of interventions in the school context is crucial (23, 24). As Jourdan et al. (24) suggest, there is an urgent need for context-sensitive approaches, intersectoral partnerships, and sustainable strategies to improve the implementation of health-promoting practices in schools. Therefore, there is a need for collaborative efforts between the education and health sectors to implement effective physical activity interventions (24). Further, understanding potential barriers and facilitators, including contextual factors to successful implementation, is vital (25), yet few studies have explored this in detail. Identifying barriers to implementing planned interventions can help create strategies to overcome these challenges (26). Involving experts, school staff, and students early in the implementation process is crucial for exploring barriers and facilitators. Their collaboration offers valuable insights into the experiences of those served by an intervention, those involved in its delivery, and the intended users of its results. Such research has been shown to generate knowledge perceived as trustworthy and relevant, helping to transfer evidence into real-world practice (27). Therefore, this study aims to explore how multiple stakeholders, including school staff, students, and experts, perceive the barriers and facilitators to implementing a school-based intervention that extends the school day with additional physical activity in Sweden.

## 2 Material and methods

### 2.1 Design

An inductive, explorative, qualitative design was used to explore the perceptions of school staff, students, and experts. Qualitative methods are valuable for gaining insight into multiple stakeholders’ views and exploring complex dynamics and issues in depth (28). Focus groups were conducted with school staff and students, and interviews were conducted with experts. The focus groups provided insights into the practical experiences and perceptions of school staff and students regarding the potential implementation of a school-based intervention that extends the school day with additional physical activity. Focus groups were selected for these participants to facilitate dynamic discussions and capture a range of perspectives in a collaborative setting. This helped identify specific barriers and facilitators from those directly involved in the school environment. The interviews with experts offered a broader view of the systematic and policy-related factors that could influence the implementation of school-based interventions. Interviews were chosen for experts to allow for in-depth explorations of their specialised knowledge and experiences in a more focused manner. These data sources provided a comprehensive understanding of the barriers and facilitators of implementing a school-based intervention that extends the school day with additional physical activity.

The study is part of the preparation phase of a cluster-randomised controlled trial. The intervention will be conducted in secondary schools in Stockholm, Sweden, targeting 8th-grade adolescents (14–15 years). Briefly, the planned intervention will involve extending the school day by adding 60 min three times per week. It will include the following components: (1) diverse types of physical activities, (2) homework support with short activity breaks, and (3) walking while listening to an audiobook.

Ethical approval for this study was obtained from the Swedish Ethical Review Authority (Dnr 2021-00911). Informed consent to participate has been obtained from all participants and the student's legal guardians.

### 2.2 Setting and participants

The schools were located in two municipalities in the Stockholm Region, with around 35,000 inhabitants, and in the Gotland Region, with around 60,000 inhabitants. The average income of the municipalities in the Stockholm Region was relatively high, while in the Gotland Region, it was average in the Swedish context (26). Schools were selected using purposive sampling based on geographic location and socioeconomic status. Specifically, schools were chosen from two municipalities in the Stockholm Region and from the Gotland Region to ensure a diverse representation of urban and rural areas with varying socioeconomic conditions. One selected school had previous experience implementing additional physical activity, while the others did not. The selection aimed to include a mix of schools to capture a broad range of experiences and perspectives.

During the recruitment for this study, invitations were emailed to the principals of 10 secondary schools. Three schools agreed to participate, while the main reason for non-participation was that the schools were dealing with the consequences of the Covid-19 pandemic. The schools that declined to participate also included those from both high and low socioeconomic backgrounds. Physical Education (PE) and other subject-specific teachers who could be involved in potential extra-curricular physical activities were invited to the focus groups by the principals in each school. The schools selected student representatives before the invitation. They were advised to invite both male and female 8th-grade students who they believed would be comfortable discussing the study's issues in a group setting with other students, teachers, and the principal. Additionally, they were encouraged to include students who were both physically inactive and active. Experts were recruited through snowball sampling and interviewed separately. One expert was employed at the municipality of Stockholm, one was employed at the Swedish National Agency for Education, and one was a pedagogical researcher. They were selected based on their experience designing and implementing school-based physical activity interventions. All invited experts agreed to participate. In total, 16 participants agreed to participate in the study. The experts were one male and two females, with a mean age of 60.3 years (range 49–69). Three mixed focus groups were conducted with a total of 13 participants, including school principals (*n* = 3), teachers (*n* = 6), and students (*n* = 4). The principals and teachers included eight females and one male with a mean age of 49.2 years (range 28–65). The students were two females and two males in 8th grade, with a mean age of 14.9 years (range 14–15).

### 2.3 Data collection

#### 2.3.1 Focus groups

The data was collected through focus groups to explore the participants' perceptions of the barriers and facilitators to implementing a school-based intervention. This allowed for an in-depth understanding of their thoughts (28). The focus groups were conducted at convenient times and locations for the participants. One focus group was conducted in person, and two were conducted online based on the participants' preferences. Each focus group lasted approximately 60 min as the participants could not allocate more time for the discussions.

All focus groups were conducted by a moderator and an observer (SA, GN, BH). The focus groups included a brief description of the purpose of the study and a preliminary plan for a planned intervention. This initial plan provided a starting point for discussion for the stakeholders to critique, modify, and build upon*.* A semi-structured focus group schedule was developed and pilot-tested in the first focus group, with no changes needed to the guide. The data from the first focus group was included in the analysis since it was planned as part of the study from the beginning. The purpose of testing the schedule was mainly to determine if any minor revisions were necessary. Even if revisions had been required, the data would still have been included in the analysis due to the semi-structured nature of the guide, which allowed for flexibility in how questions were posed. The questions included previous experiences of extra-curricular physical activity and homework support and potential barriers and facilitators to implementing the planned intervention. For example, participants were asked their thoughts on extending the school day to make room for extra teacher-led physical activity. Probing questions were used to clarify specific content or request examples of situations participants mentioned. The moderator and the observer took field notes during and after the focus groups.

#### 2.3.2 Interviews

Interviews were conducted with experts, including representatives from government agencies and researchers (*n* = 3), to explore the factors, including barriers and facilitators, focusing on implementing extra scheduled physical activity in secondary schools. The same question areas as in the focus group schedule were also used in the interviews. The questions were adapted to an interview format. Interviews were chosen to gather expert opinions rather than facilitate discussion, as would be the case with focus groups. Interviews allow for a concentrated exploration of individuals' past experiences (28) in developing or implementing school-based interventions, which may be overlooked in focus groups where it can become more generalised. The interviews were conducted at a convenient time for each participant. All interviews were conducted digitally due to the social restrictions of the Covid-19 pandemic. The interviews lasted approximately 60 min. Two of the authors (SA and GN) conducted all the interviews, which took place between March and April 2021. The interviewer took field notes during and after the interview. The data from interviews and focus groups were audio-recorded and transcribed verbatim by a professional typist. The first author (SA) checked the transcripts' accuracy. Quotes were translated from Swedish to English after the analysis was completed.

### 2.4 Data analysis

SA read through the transcripts several times to become familiar with the data. The focus was mainly on the manifest content, with a low level of interpretation, following inductive qualitative content analysis procedures (29, 30). This process helped to gain an initial understanding of areas of interest in the data. Specifically, the focus was on identifying any factor, characteristic, institutional structure, view, or belief that either hindered or facilitated students’ engagement or the implementation of extra physical activity in their school. Starting from this initial understanding, SA identified meaning units in the transcripts, words, sentences, or paragraphs containing essential aspects related to the aim of the study. These meaning units were first condensed and then labelled with codes. Then, the condensed meaning units and codes representing similar aspects were grouped into sub-categories. These sub-categories were compared and summarised into main categories. SA and LF further discussed the sub-categories and categories. All authors reviewed the categories and sub-categories to enhance the trustworthiness of the findings (30). The discussion among all the authors focussed on the content of each sub-category and category within its context. Any discrepancies were discussed until a consensus was reached among all authors.

## 3 Results

**Figure 1:**
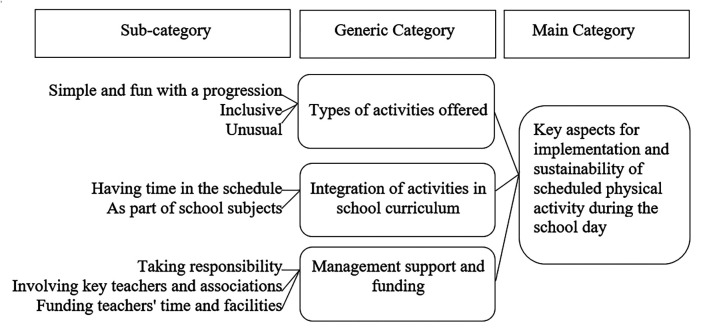
Main category, generic categories and sub-categories.

The analysis revealed one main category, three categories and eight sub-categories. The categories were: “Types of activities offered”, “Integration of activities in school curriculum”, and “Management support and funding”. The categories emphasise the barriers and facilitators to designing and implementing activities that are enjoyable, seamlessly integrated into the school's daily routine, and financially supported to ensure that teachers can effectively and sustainably implement them. The main, generic and sub-categories are presented in Figure 1.

### 3.1 Types of activities offered

#### 3.1.1 Simple and fun with a progression

To facilitate the implementation of extra physical activities and increase engagement, experts and teachers proposed introducing a variety of fitness, strength training, and physical coordination activities tailored to the adolescents' age. They also emphasised that these activities should be concrete and simple yet incorporate a progression to ensure students feel a sense of development and learning. Furthermore, the intensity of the activities should be sufficient to raise the heart rate without causing excessive sweating, as the students pointed out that they are reluctant to shower after the activities. Both teachers and students suggested incorporating pulse-enhancing activities:

They would be more motivated if it were like activities with high intensity that students enjoy. (Teacher 2, FGD1)

Most students like the high-intensity sessions. (Student 2, FGD1)

Students and teachers suggested that allowing students to choose activities would further facilitate their implementation. Additionally, teachers requested supportive materials that included suggestions for various activities.

#### 3.1.2 Inclusive

When designing activities, both school staff and experts emphasised the importance of implementing flexible activities that can be adapted to ensure that all students can participate. This includes students with disabilities, those who feel insecure, those with low self-confidence, and those who may struggle with specific exercises:

I think, I'm not worried about those who love to move and feel completely comfortable with this, but we must not lose sight of the others, because they're the ones who are really the most important. (Expert 2)

From the experts' point of view, inclusive activities were described as a prerequisite for implementation. One expert pointed out that activities should avoid stigmatising or discriminating against groups of students. These activities should benefit all students and align with the principles outlined in the Convention on the Rights of the Child—with the children's best interests in mind. School staff also discussed the practical challenges and opportunities in implementing suitable and inclusive activities. They suggested that one effective way to tailor these activities for broad participation is to avoid competitive games or sports:

The entry point is a bit up to those who are going to lead an activity, they have to think about the setup. It cannot be too competitive, it must be aimed at everyone, so that is the entry point, I think. (Principal 1, FGD 1)

#### 3.1.3 Unusual

Another suggested way to promote inclusivity was to offer activities or sports that are a little unusual or uncommon. This approach was suggested to help prevent disparities among students based on their previous knowledge or experiences of the activities. One student shared: “*Not so complicated exercises, so you don't have to have played football to participate*” (Student 3, FGD 2). It is important to provide a flexible range of activities that caters to all student's needs and interests while also appealing to those who typically do not engage in physical activity. To enhance the motivation of those students, an expert suggested focusing on activities that are exciting and require minimal effort:

And those who need the most help are those who need an entry point, how are we going to motivate all students to be physically active? Then you must look at the lowest common denominator, so that, what can suit everyone so that you reach everyone. Not only those who are already active. (Expert 2)

Paradoxically, activities requiring little effort may not be suitable for more active students. Therefore, it was suggested that more students would benefit if one offered them a choice of parallel or different activities. However, this would require more resources, especially teachers who could lead the sessions.

### 3.2 Integration of activities into the school curriculum

#### 3.2.1 Having time in the schedule

While the school staff and experts thought extra physical activity feasible, they also stressed the importance of integrating these activities into the school's schedule to encourage participation.

They expressed the need to tailor the physical activity sessions to fit each school's schedule so that the activities could occur. One expert (Expert 1), with previous experience extending the school day with extracurricular physical activities, emphasised, “*Compliance is the most important thing*” (Expert 1). Having time in the schedule for extra physical activity could help ensure that students participate and that the activity sessions are carried out as planned.

Students expressed that extending the school day with extra physical activity could be challenging. Although motivating tired students to participate may be difficult, they suggested that scheduling the activities in the morning might be more effective:

Maybe you become more awake (student 3, FGD 2)

Yes, you might perform better in school (student 4, FGD 2)

Barriers to implementing extra physical activity included scheduling activity sessions after regular school hours, which might pose challenges for students involved in extracurricular activities or who live far from the school. A principal suggested the activities to become a natural part of the schedule:

Then it's not about staying an extra hour for something specific, it's about the schedule looking different, it's a longer schedule, for everyone. (Principal 2, FGD 2)

School staff and experts emphasised the need to implement more physical activity during long theoretical lectures. They suggested incorporating activity sessions of 20–30 min, as these would be easier to integrate than longer sessions of 60 min. Schools could schedule shorter breaks between lessons or offer extra physical activity as electives or “student's choice” to condense the school day. Activity breaks between lessons were also suggested as a feasible and sustainable option.

#### 3.2.2 As part of school subjects

Connecting the physical activity sessions with students' learning and school subjects was perceived as essential to increase this understanding. For example, teachers and students saw opportunities to walk while listening to audiobooks as part of Swedish or English lessons.

You could put it in for a lesson, that you take half an hour, and we go and walk and listen. (Teacher 3, FGD 2)

Focusing on students' learning was also seen as important. Participants pointed out that it is beneficial if students and teachers understand the health-promoting and academic benefits of physical activity to be motivated to carry out the activities.

As a school, we need to be clear that this gives better results for all students. (Principal 2, FGD 2)

The participants emphasised that students could become more engaged and inspired to be physically active if they understood that it could help improve their study results. One student also mentioned that extra physical activity could help students concentrate better and ultimately achieve better grades:

It could actually increase the students' ability to work during lessons. So, you could raise your grades if you need it. (Student 1, FGD 1)

### 3.3 Management support and funding

#### 3.3.1 Taking responsibility

Having support from management was seen as crucial. Management needs to take overall responsibility, be clear, and highlight the importance of involving teachers. One expert expressed:

This thing about the school management it's probably the most important. Because unless the school management shows their full support, so that everyone understands that this is important, this is what we're going to do this at this school, it doesn't help how good the PE teachers are. (Expert 1)

The experts suggested that a contract with schools could increase participation in the research project. Another suggestion was to have focus groups involving all involved staff to introduce and plan the activities.

#### 3.3.2 Involving key teachers and associations

Physical education teachers were generally seen as pivotal due to their experience and knowledge in teaching physical activity. A principal suggested that assistant teachers could also lead physical activity sessions, as they are easy to schedule:

It is just to re-schedule an assistant teacher and say that you should hold a physical activity session (Principal 1, FGD 1).

To have at least one enthusiastic and ambitious teacher involved in implementing the activities was highlighted as key to ensuring their sustainability and continuity.

There must be someone very enthusiastic at every school who kind of takes responsibility for this being implemented. (Expert 1)

An enthusiastic teacher could also contribute to students' motivation and participation. At least one teacher would be needed to conduct the sessions, but that person could also train other teachers to lead the activities. The experts emphasised several teachers would require less time for each teacher:

So if more teachers do it, there will be less time per teacher (Expert 2)

If too few teachers are involved, it could be problematic to ensure the activities' sustainability in the long term, according to the teachers and principals. An enthusiastic teacher can become isolated when performing the activities, making it difficult to carry out the activities. It was therefore seen as essential to involve more teachers, even though some will be more engaged than others. According to a participant, it can be fun at the beginning to lead the activities, but a lack of proper support poses a risk:

What will definitely happen after a while is that there is a risk to those who carry this out, the person who leads the activities themselves, will be left alone with it at the school. And there may be a change of headmaster, there may be other staff changed around, there will be new staff who want to pursue other issues. As a result, you become very, very lonely as a PE teacher. That is very common, I would say, in my opinion. It was fun at first. (Expert 3)

Management could also consider contacting different associations to help facilitate the activity sessions. Teachers who had an experience with extended school days emphasised the importance of having physical education teachers and external leaders from different sports clubs lead the activity sessions. One key factor in promoting this idea was the establishment of alliances with local associations from the surrounding area. These alliances were described as a “win-win situation” that would benefit teachers by providing help and inspiration and associations by allowing them to showcase their activities and attract students.

#### 3.3.3 Funding teachers' time and facilities

The staff funding to lead activity sessions was described as a challenge, especially if the schools themselves needed to finance it. Questions about where the resources should come from and who would pay for teachers' time were raised, especially from the school principals:

This idea is that teachers think it's so fun that there are better results for the school that they would work an extra hour or three hours a week. This ideás not realistic. Of course, you have your duties that you should do, so it must also be integrated into your [paid] work. (Principal 2, FGD 2)

The school staff, both teachers and principals, agreed that extra resources would be needed to integrate additional physical activities in the schedule. They explicitly asked: *where should the resources come from (Teacher 4, FGD 3).* However, the school staff also saw opportunities that, if planned correctly, the activities do not need to be so costly.

The school staff also raised concerns about the cost and availability of facilities, like local sports halls, for hosting activities. Several teachers described it as the “greatest dilemma”. The principals explained that booking times at these facilities could be challenging due to high demands from other schools and sports clubs. On the other hand, many participants suggested that activities could be held in classrooms, the schoolyard, or other outdoor areas. However, the experts emphasised that being outside in the winter may not always be popular among students and staff.

I think it will be hard enough to get them [students] to walk, like when it's cold and snowy and wet outside. (Expert 1)

Walking in winter could be a great way to get out and move, but it could also be challenging to motivate students when it's cold. Considering the weather when planning outdoor activities was emphasised, especially given that Sweden has a long winter.

## 4 Discussion

### 4.1 Result discussion

This study explored how multiple stakeholders, including school staff, students, and experts, perceive the barriers and facilitators for implementing a school-based intervention that extends the school day with additional physical activity in Sweden. The results were presented in three categories: (1) “types of activities offered”, which emphasised the importance of designing activities that are fun, inclusive and unusual; (2) “integration of the activities into school curriculum”, which concerned the sustainability of the intervention by incorporating physical activity throughout the school day and across school subjects, and (3) “management support and funding” that referred to the financial resources and facilities needed to support teachers who leading the activities, ensuring a successful implementation.

The results show that schools can design engaging physical activities that are simple, fun, unusual, and inclusive. These activities should align with students’ interests and preferences to encourage participation. Activities with a low entry point were also perceived to encourage those with low self-confidence or difficulties to participate. Previous research has also shown the benefits of individual/team development rather than competition in promoting physical activity (17). Similarly, a systematic review by Martins et al. (31) found that having fun was the most frequently mentioned factor encouraging physical activity among adolescents. It emphasised the need for diverse, challenging activities without student competition. Most studies in the review (31) focused on adolescent girls and their perspectives on factors influencing their participation in physical activity, rather than on specific interventions. The current study involved engaging stakeholders during the intervention's planning phase to incorporate the perspectives of those being served by a planned intervention. This is essential for tailoring interventions to local contexts and enhancing implementation. Integrating diverse stakeholder input at this stage can reveal potential barriers, as most school-based physical activity studies typically address these perspectives only after the intervention has begun.

Involving several teachers and providing them with adequate training and support was found as essential for successful implementation. Another important facilitator presented in the result was the possibility of integrating physical activities into the school schedule, combining physical activity with lessons in various school subjects (e.g., walking while listening to an audiobook required in the Swedish, English or History class). Several studies have raised the importance of considering the complexity of the school environment and the need to tailor the implementation of initiatives to these specific contexts (16, 17, 23). Jago et al. argue for a shift from uniform interventions to more flexible, context-specific approaches that allow schools to adapt the content of physical activity programmes to their unique circumstances. However, there is limited evidence on how to make these adjustments effectively. Previous systematic reviews found that involving staff in the provision of physical activity enhances implementation in school settings (32, 33). The findings in this study highlighted that staff involvement is crucial for raising awareness about the health benefits of physical activity, particularly in relation to students' academic performance. This increased awareness may motivate teachers to integrate physical activity into their subjects. To promote this awareness, the planned intervention will include workshops for teachers aimed at customising the implementation to fit each school's context and promote understanding of the planned initiative.

For staff to be involved in delivering a planned intervention, it seems crucial to ensure management support and funding of staff hours and facilities to carry out the additional physical activity. To some extent, these findings echo the barriers to implementation found in healthcare professionals working with increasing physical activity among cardiac patients, where support from administration and leadership was considered key (34). This is also consistent with a recent study about teachers' perspectives of the barriers and facilitators to the delivery of a school-based physical activity intervention (35), which found that some teachers refused to participate due to time constraints which may be linked to a lack of managerial prioritisation of physical activity activities. Taken together, the results of this study highlight the importance of involving stakeholders' perspectives in designing physical activity interventions to tailor the intervention to each school setting.

### 4.2 Strengths and limitations

Data was collected from different stakeholder groups (e.g, students, staff, and experts), which increased the study's credibility (36, 37). Focus groups and interviews enabled the triangulation of methods (38) and captured multiple perspectives. Using an interview guide with semi-structured questions also allowed the participants to express their experiences (39). However, it is possible that all groups did not feel they could openly express their thoughts in the focus groups due to power dynamics.

Credibility was enhanced through several strategies. Researcher triangulation was conducted in the data collection and analysis, increasing the study's credibility (36, 37). The use of multiple data sources (students, school staff and experts) and methods (focus groups and interviews) provided a comprehensive view of the phenomena under study. Member checking was not used, and it could have provided new insights if it had been conducted. Dependability was ensured by well-documenting the data analysis process and reflecting on the researchers' pre-understanding to create awareness and ensure that data analysis focused on the participants' perceptions and previous experiences. An audit trail was maintained, detailing each step of the research process. Furthermore, the researchers continuously reflected upon their potential pre-conceptions to ensure confirmability and that the findings were based on the participant´s voices.

Although a relatively small number of participants were included, they represented varied experiences and school contexts. Yet, the number of participants may limit the study's transferability. The short time for data collection, with only one hour for each interview and focus group, did not allow for a thorough description of the context and participants. Having a more comprehensive description of these aspects would have enhanced the study's transferability to other settings (37). It is a limitation that schools were recruited during the COVID-19 pandemic since many schools expressed an interest in the subject of the study but declined participation due to a heavy workload. Data collection continued until saturation was reached, but it is possible that new aspects could have emerged with a larger study population.

## 5 Conclusions

When planning a physical activity intervention in a school setting, it is important to understand students' preferences for physical activity to enhance their motivation. Schools can design physical activities that are simple, fun, unusual, and inclusive to engage all students. By aligning the activities with studentś interests and preferences, schools can encourage greater participation.

Implementation can be facilitated if several teachers deliver the activities and receive adequate training, and support will also facilitate implementation. Administrative support is also crucial; school managers should provide necessary resources, including scheduling adequate time for physical activities, integration in diverse subjects (scheduling), funding for teachers (paid working hours), and appropriate facilities.

Adapting to local needs is recommended to facilitate the implementation of the planned intervention, as the complexity of school systems requires flexibility in changing group dynamics and contexts (23). Beyond these barriers, additional extra-curricular physical activity can be a novel way to increase adolescents' physical activity levels and contribute to reducing health inequalities by including all students. The findings of this study can inform educational policies and school interventions.

## Data Availability

The raw data supporting the conclusions of this article will be made available by the authors, without undue reservation.
